# The Land of Montezuma

**Published:** 1896-11

**Authors:** 


The Land of Montezuma.—Every one who has ever read "The Conquest of Mexico," must have a desire to see that wonderful country, the land of perpetual summer, where nature wears her richest and most gorgeous attire. There the desert literally "blossoms like the rose;" there, with rare and curious forms of vegetation, the land is literally ablaze of glory. It is the land of the cactus and the orchid, the home of the orange, the tamarind and the fig; a land redolent of romance and history. Who does not want to see the old city, the scenes of such barbaric splendor during the reign of Montezuma, yet eloquent of a former civilization that was young, perhaps, when that of Egypt was born?—the scenes of the daring Spaniards' almost superhuman valor, stimulated by the thirst for gold, not glory? It is said that many points made famous by that siege and conquest are still shown to tourists; a part of the causeway, for instance, still exists, where the desperate attempt of the Spaniards to escape occurred, and where Alvarado made his famous leap for life, and part of the great park, where the wild beasts were kept, which served, not to "make a Roman holiday," but to eat such of the population as were found to be "bad Injuns," as well as captives not fit to offer to the big, goggle-eyed idols they called "gods."

To all who would like to see Mexico,—as Mr. Pecksniff used to remark, "an eligible opportunity now offers." The great Pan American Medical Congress affords the opportunity, and the great Laredo Route, the Mexican National Railroad, the means and facilities for doing so with ease, elegance, economy and comfort. The Congress will meet in the City of Mexico on the 16th of November (inst.) and sit four days. The Mexican National Railway Co., with characteristic enterprise and sagacity, has arranged to carry delegates and their families at a low rate of fare for the round trip, with privilege of stop-over at numerous points of interest, and has arranged also for side trips to other points, coincidentally. Delegates and their friends arriving at Laredo, the gateway to the Land of Wonders, will find in waiting splendid trains of vestibule cars, Pullman Palace Buffet Sleepers, in care of courteous conductors; and the road's chief surgeon, the handsome, genial and popular Dr. R. H. L. Bibb,—whom every Texan knows, will take personal charge of the excursion; and that means a good time. The cars on this route, under the supervision of a surgeon of international reputation as a sanitarian, could not be otherwise than "hygienic;" they are clean and well ventilated. At several points on the route are eating heuses whose *cuisine* has acquired fame far and near, where those who prefer not to dine on board can get something good to eat, American style.

This road takes the tourist through the loveliest parts of that wonderful scenic country. It is a veritable living panorama; the road rising at times to an altitude of 10,000 feet above the level of the sea, thence descending to wide stretches of valley of phenomenal verdure and fertility, where the strawberry and the melon flourish in perennial deliciousness. One most comforting fact in connection with this road is, never, within its history, has a passenger ever been injured; accidents are unknown, rendered next to impossible by a never-sleeping vigilance and watchful care for the comfort and safety of the traveling public. The route passes through the battle fields of Monterey and Buena Vista, where American volunteer troops, under Scott and Tayloı, won imperishable fame. Tourists have opportunity of seeing also the celebrated Hot Springs of Mexico, the Topo Chico Springs, and also the City of San Luis Potosi, in the heart of the mining district whence, in all probability, old Montezuma derived his stores of gold galore that so tempted the Spaniards to his undoing.

It would be hard to imagine a more glorious trip than such as is here offered, and that, too, at such little expense that anybody can afford it. The hotel rates are about half as much as in this country.

But it is not alone as a pleasure trip that it is desirable; one can kill two birds, etc. No country in the world offers better inducements and opportunities for advantageous speculative investment. The mineral and agricultural resources have barely been tapped, and it will not be long ere American enterprize and capital will develop them, and now is the time to get in on the ground flor.

Be careful to see that your ticket reads via Laredo Route, Mexican National Railway, via San Luis Potosi. See advertisement in this issue.

